# Risk of medication overuse headache across classes of treatments for acute migraine

**DOI:** 10.1186/s10194-016-0696-8

**Published:** 2016-11-24

**Authors:** Kristian Thorlund, Christina Sun-Edelstein, Eric Druyts, Steve Kanters, Shanil Ebrahim, Rahul Bhambri, Elodie Ramos, Edward J. Mills, Michel Lanteri-Minet, Stewart Tepper

**Affiliations:** 1 Department of Clinical Epidemiology & Biostatistics, McMaster University, Hamilton, ON Canada; 2 Redwood Outcomes, 302-1505 2nd Ave. West, Vancouver, BC Canada; 3 Department of Medicine, St. Vincent’s Hospital, The University of Melbourne, Melbourne, Australia; 4 Department of Medicine, Faculty of Medicine, University of British Columbia, Vancouver, BC Canada; 5 School of Population and Public Health, Faculty of Medicine, University of British Columbia, Vancouver, BC Canada; 6 Pfizer Ltd, New York, NY USA; 7 Pain Department, CHU Nice, France - FHU InovPain, Université Nice Côte d’Azur, Nice, France; 8 Geisel School of Medicine at Dartmouth, Hanover, NH USA; 9 INSERM U1107, Neuo-Dol, Trigeminal Pain and Migraine Université Auvergne, Clermont-Ferrand, France

## Abstract

**Background:**

The most commonly prescribed medications used to treat migraine acutely are single analgesics, ergots, opioids, and triptans. Due to varying mechanisms of action across drug classes, there is reason to believe that some classes may be less likely than others to elicit Medication Overuse Headache (MOH) than others. We therefore aimed to determine whether certain classes of acute migraine drugs are more likely to elicit MOH than others.

**Methods:**

A comprehensive systematic literature was conducted to identify studies of varying designs that reported on MOH within the considered treatment classes. Only studies that reported MOH according to the International Classification of Headache Disorders (ICHD) were considered. Since no causal comparative design studies were identified; data from prevalence studies and surveys were retrieved. Prevalence-based relative risks between treatment classes were calculated by integrating both medication overuse and medication use from published studies. For each pair wise comparison, pooled relative risks were calculated as the inverse variance weighted average.

**Results:**

A total of 29 studies informed the relative risk between treatment classes, all of which reported country-specific data. Five studies reported country-specific medication use data. For triptans versus analgesics the study relative risks generally favored triptans. The pooled relative risk was 0.65 (i.e., relative risk reduction of 35 %). For ergots versus analgesics, a similar trend was observed in favor of ergots with a relative risk of 0.41. For triptans versus ergots, the direction of effect was mixed, and the pooled relative risk was 1.07. Both triptans and ergots appeared favorable when compared to opioids, with pooled relative risks of 0.35 and 0.76, respectively. However, the evidence was limited for these comparisons. Analgesics and opioids also appeared to yield similar risk of MOH (pooled relative risk 1.09).

**Conclusion:**

Our study suggests that in patients receiving acute migraine treatment, analgesics and opioids are associated with a higher risk of developing MOH compared with other treatments. These findings provide incentive for better monitoring of use of analgesics and opioids for treating acute migraine, and suggest possible clinical preference for use of so-called “migraine-specific” treatments, that is, triptans and ergots.

**Electronic supplementary material:**

The online version of this article (doi:10.1186/s10194-016-0696-8) contains supplementary material, which is available to authorized users.

## Background

Medication-overuse headache (MOH) is caused by overuse of medications for migraines or other pain disorders. According to the International Classification of Headache Disorders, 3^rd^ Edition, Beta (ICHD-3), MOH is defined as headache occurring on 15 or more days per month developing as a consequence of regular overuse of acute or symptomatic headache medication (on 10 or more, or 15 or more days per month, depending on the medication) for more than 3 months [1].

MOH manifests as increased frequency and intensity of headaches or migraine attacks and enhanced sensitivity to stimuli that elicit these episodes [2] Although the mechanisms underlying MOH are not fully elucidated, it is hypothesized that repeated medication use could elicit increased headache attacks as a consequence of neuronal plasticity that may increase responsiveness to triggers. The prevalence of MOH is 1–2 % in the general population worldwide, and because of the estimated socio-economic cost, it is likely to be the most costly neurological disorder known [3–6].

Commonly prescribed medications for migraines may include analgesics, ergots, opioids, and triptans. Due to varying mechanisms of action across drug classes, there is reason to believe that some classes may be less likely than others to elicit MOH. Because of the estimated socio-economic burden of MOH, it is therefore important to establish which drug class generally is least likely to elicit MOH.

The aim of this study was therefore to determine whether certain classes of acute migraine drugs are more likely to elicit MOH than others. To achieve this, we performed a comprehensive systematic literature review of available evidence and, to the extent data allowed, extrapolated the comparative risks of MOH associated with available drug classes.

## Methods

### Eligibility criteria

Eligible studies could be either observational or clinical (randomized or non-randomized) in nature. Only studies that included adults 18 years of age and older who were suffering from acute migraine were eligible. Eligible studies must have reported MOH by treatment class (i.e. analgesic, ergot, opioid, or triptan), and according to versions of ICHD-2 [1, 7–9].

### Search strategy

In consultation with an academic medical librarian, we conducted a systematic search of the medical literature using MEDLINE, EMBASE, and the Cochrane Controlled Trials Register (from inception to March 24, 2014). The search strategy was sensitive and broad, consisting of the following: ‘medication overuse headache’ and ‘migraine’. Conference abstracts provided through the EMBASE search were also reviewed to determine if there were relevant studies recently completed. Additionally, hand searches of the bibliographies of published systematic reviews and health technology assessments were performed. All searches were performed independently, in duplicate.

### Data extraction

We extracted data on the total number of patients, the number of patients with MOH by treatment class (i.e. analgesics, ergots, opioids, and triptans), MOH diagnostic criteria, and the country/region of each study. These data were extracted from the baseline characteristics of the studies. Two reviewers independently extracted and recorded all data in a Microsoft Excel spreadsheet. All data extraction were then checked by a third reviewer.

### Materials

Out of 443 abstracts reviewed, a total of 29 studies were eligible [10–39]. Additional file 1: Figure S1 shows the flow chart, and Additional file 1: Table S1 show the list of studies excluded following full-text review with accompanying reason for exclusion. Table 1 presents the characteristics of the included studies. All included studies had been published since the year 2004, when the ICHD-2 was first released. Eleven of the included studies adhered to the definition of MOH in version 1 of the ICHD-2, whereas 9 studies adhered to the revision put forward in 2005, and 9 studies adhered to the appendix put forward in 2006. No studies made use of ICHD-3, released in beta in 2013. All studies took place in Europe (1 in Denmark, 3 in France, 2 in Germany, 19 in Italy, 2 in Norway, 1 in Spain, and 1 in Sweden). Two studies were population based, the remaining were based out of a clinical setting (i.e., investigator headache centers, hospital departments of neurology, of headache and pain clinics).

**Table 1:** Summary of study characteristics

Study	Country	Diagnosis classification	Study setting	No. patients	No. medication overuse headache			
					Analgesics	Ergots	Opioids	Triptans
Altieri et al. 2009 [11]	Italy	ICHD-2 [7]	Clinic	27	11	NR	NR	4
Ayzenberg et al. 2008 [12]	Germany	ICHD-2 [7]	Clinic	29	14	NR	NR	15
Biagianti et al. 2012 [13]	Italy	ICHD-2 Revised [8]	Clinic	52	26	NR	NR	20
Boe et al. 2007 [14]	Norway	ICHD-2 Appendix [7]	Clinic	100	20	1	NR	23
Coppola et al. 2010 [15]	Italy	ICHD-2 Appendix [7]	Clinic	29	10	NR	NR	9
Cupini et al. 2009 [16]	Italy	ICHD-2 Appendix [7]	Clinic	33	NR	1	NR	4
Di Lorenzo et al. 2009 [17]	Italy	ICHD-2 [7]	Clinic	107	18	NR	NR	29
Donnet et al. 2009 [18]	France	ICHD-2 [7]	Population	320	157	25	29	64
Dousset et al. 2013 [19]	France	ICHD-2 Appendix [7]	Clinic	42	8	1	0	9
Galli et al. 2011 [20]	Italy	ICHD-2Appendix [7]	Clinic	82	21	2	3	22
Gambini et al. 2013 [21]	Italy	ICHD-2 Revised [8]	Clinic	63	33	NR	NR	21
Gomez-Beldarrain et al. 2011 [22]	Spain	ICHD-2Revised [8]	Clinic	42	25	NR	NR	3
Hagen et al. 2009 [23]	Norway	ICHD-2 Appendix [7]	Clinic	56	18	NR	14	17
Jonsson et al. 2011 [6]	Sweden	ICHD-2 Appendix [7]	Population	799	517	7	33	66
Lorenzo et al. 2012 [24]	Italy	ICHD-2 Appendix [7]	Clinic	43	17	NR	NR	8
Pageler et al. 2008 [25]	Germany	ICHD-2 [7]	Clinic	20	1	3	NR	5
Perrotta et al. 2010 [26]	Italy	ICHD-2 [7]	Clinic	31	11	NR	NR	19
Perrotta et al. 2012 [27]	Italy	ICHD-2 [7]	Clinic	27	4	NR	NR	6
Radat et al. 2013 [28]	France	ICHD-2 [7]	Clinic	17	2	NR	2	4
Rainero et al. 2006 [29]	Italy	ICHD-2 [7]	Clinic	18	NR	2	NR	3
Relja et al. 2006 [30]	Italy	ICHD-2 [7]	Clinic	101	38	9	0	12
Rossi et al. 2006 [31]	Italy	ICHD-2 Revised [8]	Clinic	118	63	3	NR	24
Rossi et al. 2011 [32]	Italy	ICHD-2 Revised [8]	Clinic	100	57	1	NR	23
Sances et al. 2010 [33]	Italy	ICHD2 Revised [8]	Clinic	172	42	5	5	50
Sandrini et al. 2011 [34]	Italy	ICHD-2 Appendix [7]	Clinic	56	23	2	NR	20
Terrazzino et al. 2010 [35]	Italy	ICHD-2 Revised [8]	Clinic	227	79	2	1	32
Trucco et al. 2010 [36]	Italy	ICHD-2Revised [8]	Clinic	70	18	0	NR	9
Valguarnera et al. 2010 [37]	Italy	ICHD2 [7]	Clinic	95	20	2	2	30
Zeeberg et al. 2006 [38]	Denmark	ICHD2 Revised [8]	Clinic	216	63	8	12	43

In general, the included studies reported on use of treatment classes, but did not distinguish which individual agent or agents (e.g., sumatriptan or eletriptan for triptans) had been administered to patients. Thus, analysis by individual agents was not possible. However, since all eligible studies were published between 2006 and 2013, it is reasonable to assume that most patients receiving ergots received dihydroergotamine (DHE) and not the older ergotamine tartrate.

All studies reported MOH as prevalence estimates within included study population; no study reported MOH as an outcome. All included studies were either observational (prospective or retrospective), clinical cohorts, or population surveys.

### Data considerations

As indicated in section 3.4, no trials or observational studies looking at multiple migraine interventions reported development of MOH as an outcome. For this reason, the comparison between interventions had to be based on studies reporting prevalence estimates of MOH. While relatively few available studies were specifically designed to estimate prevalence, several still provided data on the proportion of patients using each of the interventions that had developed MOH. For example, several survey studies reported in their baseline table the number and proportion of enrolled patients with MOH. Other publications described the characteristics of migraine patients in one or more clinics, which included the number and proportion of patients with MOH. To justify inclusion of studies not designed to estimate prevalence, we made the assumption that prevalence estimates were not confounded by the designs of these studies. We verified that the eligibility criteria in these studies did not include criteria related to MOH.

Since the prevalence of MOH is highly correlated with the prevalence of drug dispensing, prevalence estimates do not provide a good basis for comparison of risk of MOH associated with the different interventions. For example, triptans are prescribed more frequently than ergots, and so we expect to see higher numbers of triptan-related MOH occurrences than ergot-related MOH occurrences. To form a fair basis for comparisons of the relevant interventions, it is therefore also necessary to know the frequency of treatment use. These were estimated post-hoc by a systematic review of the literature, and were incorporated in the calculations of comparative risk of MOH. To this end, we systematically searched MEDLINE and EMBASE for population-based studies providing estimates of medication use prevalence for the countries/regions represented in the eligible MOH prevalence studies.

The prevalence of medication use for migraine was derived from 5 studies identified in our literature review (See Table 2) [23, 40–43]. Because the data on medication prevalence use was limited, some assumptions needed to be made. For all countries, the prevalence of opioid use among those with migraine was not available. The closest evidence of opioid use prevalence that we identified was a Swedish study including chronic headache patients. This study yielded an opioid use prevalence of 4.1 %. Since use of opioids is known to be low in Europe (which is where all included studies came from) and since we believed it reasonable to assume opioid use among chronic daily headache patients would likely be higher than opioid use among acute migraine patients, we assumed an opioid use prevalence of 2 % for all included studies.

**Table 2:** Prevalence of medication use for episodic migraine in Europe (column 2) and the applied adjustment factors in calculating MOH prevalence and risk ratios (columns 3–6)

Country/Region	Drug class (Prevalence, %)	Analgesics	Ergotamines	Opioids	Triptans
Denmark [41]	Analgesics (NA)	--	NA	NA	NA
	Ergotamines (NA)	NA	--	NA	NA
	Opioids (2.0)	NA	NA	--	1:13 (0.08)
	Triptans (26.0)	NA	NA	13:1 (13.0)	--
France [40, 42]	Analgesics (12.0)	--	4:1 (4.00)	6:1 (6.0)	6:10 (0.58)
	Ergotamines (3.0)	1:4 (0.25)	--	3:2 (1.50)	1:7 (0.14)
	Opioids (2.0)	1:6 (0.17)	2:3 (0.67)	--	1:10 (0.10)
	Triptans (20.8)	10:6 (1.73)	7:1 (6.93)	10:1 (10.4)	--
Germany [40, 42]	Analgesics (31.0)	--	9:2 (4.43)	15:1 (15.0)	2:1 (2.14)
	Ergotamines (7.0)	2:9 (0.23)	--	7:2 (3.50)	1:2 (0.48)
	Opioids (2.0)	1:15 (0.06)	2:7 (0.29)	--	1:7 (0.14)
	Triptans (14.5)	1:2 (0.47)	2:1 (2.07)	7:1 (7.25)	--
Italy [40, 42]	Analgesics (12.0)	--	12:3 (3.75)	6:1 (6.00)	4:5 (0.79)
	Ergotamines (3.2)	3:12 (0.27)	--	8:5 (1.60)	1:5 (0.21)
	Opioids (2.0)	1:6 (0.17)	5:8 (0.63)	--	1:8 (0.13)
	Triptans (15.1)	5:4 (1.25)	5:1 (4.72)	8:1 (7.55)	--
Norway [43]	Analgesics (NA)	--	NA	NA	NA
	Ergotamines (NA)	NA	--	NA	NA
	Opioids (2.0)	NA	NA	--	1:19 (0.05)
	Triptans (37.0)	NA	NA	19:1 (18.5)	--
Spain [40]	Analgesics (16.4)	--	4:5 (0.82)	8:1 (8.20)	1:2 (0.56)
	Ergotamines (20.0)	5:4 (1.22)	--	10:1 (10.0)	2:3 (0.69)
	Opioids (2.0)	1:8 (0.12)	1:10 (0.10)	--	1:15 (0.07)
	Triptans (29.1)	2:1 (1.77)	3:2 (1.46)	15:1 (14.6)	--
Sweden [23]	Analgesics (NA)	--	NA	NA	NA
	Ergotamines (NA)	NA	--	NA	NA
	Opioids (2.0)	NA	NA	--	3:19 (0.16)
	Triptans (26.0)	NA	NA	19:3 (6.34)	--

The medication use prevalence estimates presented in parenthesis next to treatment classes in column 2 are taken from included prevalence literature. The ratios (e.g. 3:1) presented in columns 3–6 are approximate ratios of prevalence of use of one medication over another. These ratios are also the adjustments factors multiplied to the unadjusted ratios of MOH for each study to account for the missing information about patients at risk on each medication within each study

### Analysis

For each study we first calculated the proportion of patients with MOH in each treatment class, using the total number of patients as the denominator for both proportions. Subsequently the relative risk was calculated between each pair of treatment classes. As mentioned in the previous section, these relative risks are highly driven by the proportion of patients that received each treatment. For this reason, we applied an adjustment to the relative risk estimates. In particular, we first estimated the ratio with which treatments are being prescribed in clinical practice from prevalence estimates of medication use, and multiplied the inverse of this ratio to the above-obtained relative risks (see Table 2). Further, sensitivity analysis assuming an 8 % medication use prevalence for opioids, was conducted.

The adjusted study relative risks were pooled for each comparison in a fixed-effect meta-analysis. A fixed-effect model was used to provide a fair weighted average of studies. The *meta* function in *R.v.3.0* was used to pool results and produce forest plots. While this function by convention produces 95 % credible intervals, one should only focus on the relative risk estimates and the weighted (pooled) average relative risk, since confidence intervals address sampling error and therefore are not valid under the above adjustments.

## Results

### Triptans versus analgesics

**Fig. 1:**
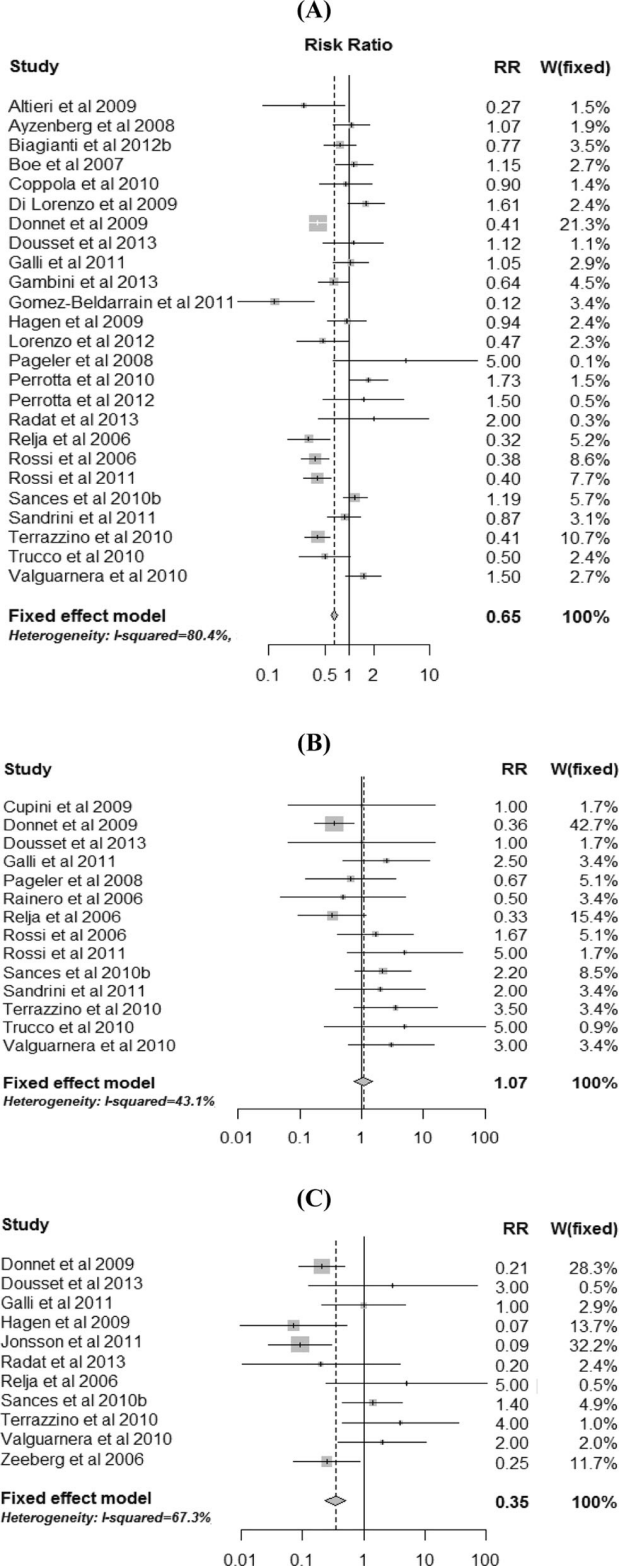
Forest plots and weighted average estimate for the relative risk of MOH for the three comparisons: **a** triptans versus analgesics; **b** triptans versus ergots; and **c** triptans versus opioids

Twenty-five studies informed MOH for both triptans and analgesics in countries where medication use prevalence estimates were also available for both. Adjusted relative risks from these studies are presented in Fig. 1a. Fourteen studies yielded relative risks in favor of triptans, with adjusted relative risks varying from 0.12 to 0.94. In 9 of these 14 studies the relative risk was statistically significant. Eleven studies yielded adjusted relative risks in favor of analgesics, with adjusted relative risk estimates varying from 1.05 to 5.00. The fixed-effect weighted average adjusted relative risk was 0.65, thus suggesting an average 35 % relative risk reduction of MOH associated with triptans compared with analgesics.

### Triptans versus ergots

Fourteen studies informed MOH for both triptans and ergots in countries where medication use prevalence estimates were also available for both. Adjusted relative risks from these studies are presented in Fig.﻿1b﻿﻿. Four studies yielded adjusted relative risk estimates in favor of triptans, two yielded no difference (i.e. relative risk of 1.00), and eight studies yielded adjusted relative risk estimates in favor of ergots. The fixed-effect weighted average relative risk was 1.07.

**Fig. 2:**
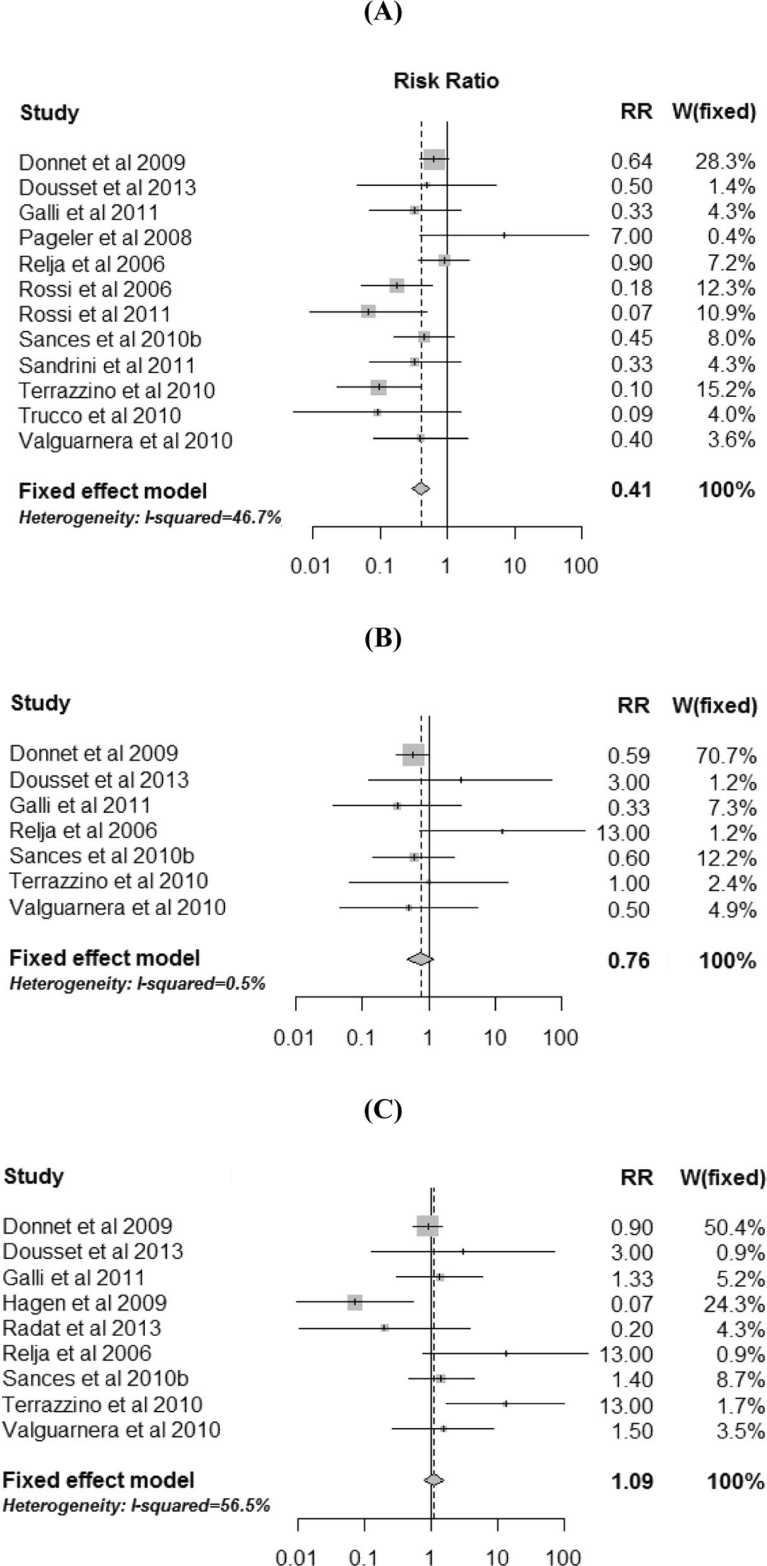
Forest plots and weighted average estimate for the relative risk of MOH for the three comparisons: **a** ergots versus analgesics; **b** ergots versus opioids; and **c** analgesics versus opioids

### Triptans versus opioids

Eleven studies informed MOH for both triptans and opioids in countries where medication use prevalence estimates were also available for both. Adjusted relative risks from these studies are presented in Fig.1c﻿. Five studies yielded adjusted relative risk estimates in favor of triptans, one study suggested no difference, and five studies yielded relative risk estimates in favor of opioids. The fixed-effect weighted average adjusted relative risk was 0.35, suggesting an average 65 % relative risk reduction of MOH with triptans compared with opioids.

### Ergots versus analgesics

Twelve studies informed MOH for both ergots and analgesics in countries where medication use prevalence estimates were also available for both. Adjusted relative risks from these studies are presented in Fig.2a. Eleven of these yielded adjusted relative risk estimates in favor of ergots, and only one small study cell with a zero cell in the analgesics arm yielded a relative risk estimate in favor of analgesics. Among the former 11, adjusted relative risks varied between 0.07 and 0.90. The fixed-effect weighted average adjusted relative risk was 0.41, suggesting an average 59 % relative risk reduction of MOH with ergots compared with analgesics.

### Ergots versus opioids

Seven studies informed MOH for both ergots amines and opioids. Adjusted relative risks from these studies are presented in Fig.2b. Four of these studies yielded adjusted relative risk estimates in favor of ergots (ranging from 0.33 to 0.60), one yielded an adjusted relative risk estimate of 1.00 (i.e., no difference), and two studies, both with zero cells in the opioids arm, yielded relative risk estimates in favor of opioids. The fixed-effect weighted average relative risk was 0.76, suggesting an average 24 % relative risk reduction of MOH with ergots compared with opioids. As noted, this likely reflects mostly DHE use rather than ergotamine tartrate.

### Analgesics versus Opioids

Nine studies informed MOH for both analgesics and opioids. Adjusted relative risks from these studies are presented in Fig.2c. Three studies yielded adjusted relative risk estimates in favor of analgesics, and six studies yielded relative adjusted risk estimates in favor of opioids. The fixed-effect weighted average adjusted relative risk was 1.09, suggesting an average 9 % increased risk of MOH with analgesics compared with opioids.

## Discussion

Our analysis aimed to evaluate rates of MOH depending on the type of medication used. We found a considerably higher rate of MOH associated with analgesics in comparison to triptans and ergots. Our findings also suggest that opioids are either associated with a higher or similar risk of MOH compared to triptans and ergots, but evidence was more limited for these comparisons. These findings should be of interest to patients, clinicians, and policy-makers as many patients may self-medicate, and the magnitude of analgesic use is potentially higher than what has generally been observed in population-based studies.

There are strengths and limitations to our analysis that should be considered. Strengths of this study include our extensive searching to complete the largest and first systematic review of MOH associated with migraine pharmacotherapies. We used an approach that integrated evidence on medication overuse and medication use, the first such effort of which we are aware.

The employed approach further strengthens comparisons between interventions, since all studies provide data on at least two interventions, and so allows for the analysis to retain within-study validity. Studies included had to use the ICHD-2 criteria or later, and thus removed most uncertainty about the appropriateness of the MOH definition, which was particularly variable in older studies. However, this eligibility criterion also came with the limitation that the American Migraine Prevalence and Prevention (AMPP) study was not eligible [35]. In fact, no US studies were eligible under the employed criteria. Therefore, generalizability of our findings to the US population is somewhat limited.

We relied instead on observational studies reporting a mix of pseudo-risk data and medication use data to approximate the relative risk between interventions. The analytical approached employed to synthesize results from these data relied on assumption that are arguably strong. There may therefore be controversy as to where the evidence fits in the evidence hierarchy. Recognizing the challenges of conducting these evaluations, and given the consistency of our study findings, the investigators believe this systematic review of observational studies provides strong inferences about the causative factors of MOH. The heterogeneity between studies was generally low, in spite of the observational nature of the included studies and the additional uncertainty one might expect from the employed medication use prevalence adjustments. Also, the findings of the individual pair-wise meta-analysis added up ‘indirectly’. For example, the direct comparison of triptans and ergots showed similar risk of MOH, and both drug classes had similar relative risk estimates when compared with analgesics (0.65 and 0.41). These consistencies thus add considerable confidence to the findings of the analyses.

There are several possible reasons for our finding increased MOH associated with analgesic and opioid use and less so with triptans and ergots. Analgesics and opioids typically work via targeting pain receptors. On the other hand, both triptans and ergots share serotonergic agonist activity and are vasoconstrictors. Furthermore, analgesics are frequently used in an over-the-counter manner whereby patients self-administer and may do so without the supervision of a clinician. Triptans are, for the most part, prescription medications, and overuse may be better monitored than analgesics. In those countries in which triptans are available without a prescription (e.g. UK, Germany), quantity limits may prevent tendency to overuse.

While our findings are in line with current clinical guidelines and prejudice in favor of targeted migraine-specific pharmacotherapy, they should still only be interpreted as exploratory due to their observational nature. Further, the inclusion of only European studies limits our ability to extrapolate the findings to other global regions.

In summary, our study suggests that in patients with acute episodic migraine, the rate of MOH associated with analgesics and opioids is considerably higher than the rate of MOH associated with triptans and ergots. These findings should be of interest to patients, clinicians, and policy-makers, as many patients self-medicate, and the magnitude of analgesic use is potentially higher than what has generally been observed in population-based studies.

## Additional file

Additional file 1: Figure S1. Flow diagram for study selection. **Table S1** List of studies excluded after full text review and accompanying reason for exclusion. (DOCX 113 kb)
