# Supplementary material for: Risk of medication overuse headache across classes of treatments for acute migraine
Source: J Headache Pain. 2016 Nov 24;17(1):107. doi: 10.1186/s10194-016-0696-8 (PMC5121112; doi:10.1186/s10194-016-0696-8)
Supplement: Additional file 1: Figure S1. — Flow diagram for study selection. Table S1 List of studies excluded after full text review and accompanying reason for exclusion. (DOCX 113 kb) [file 10194_2016_696_MOESM1_ESM.docx]

**Figure A. Flow diagram for study selection**

**Table A List of studies excluded after full text review and accompanying reason for exclusion**

| **Study** | **Reason for exclusion** |
| --- | --- |
| **Bellei et al 2012^1^** | Cohort duplicate |
| **Bellei et al 2013^2^** | Cohort duplicate |
| **Biagianti et al 2012^3^** | Cohort duplicate |
| **Cevoli et al 2010^4^** | Data not stratified by treatment class |
| **Diener et al 2007^5^** | No treatment prevalence data |
| **Ferraro et al 2012^6^** | No possible comparisons |
| **Ghiotto et al 2009^7^** | Cohort duplicate |
| **Grande et al 2009^8^** | Chronic daily headache |
| **Grande et al 2011^9^** | Chronic daily headache |
| **Imai et al 2007^10^** | No treatment prevalence data |
| **Lundqist et al 2011^11^** | Chronic daily headache |
| **Lundqist et al 2012^12^** | Chronic daily headache |
| **Munksgaard et al 2012^13^** | Chronic daily headache |
| **Paemeleire et al 2006^14^** | Cluster headache |
| **Peres et al 2007^15^** | No treatment prevalence data |
| **Riederer et al 2012^16^** | Data not stratified by treatment class |
| **Rossi et al 2008^17^** | Cohort duplicate |
| **Sances et al 2010^18^** | Cohort duplicate |
| **Zappaterra et al 2011^19^** | No possible comparisons |
| **Zidverc-Trajkovic et al 2007^20^** | No treatment prevalence data |

**REFERENCES FOR STUDIES EXCLUDED IN FULL TEXT REVIEW**

1. Bellei E, Cuoghi A, Monari E, Bergamini S, Fantoni LI, Zappaterra M, Guerzoni S, Bazzocchi A, Tomasi A and Pini LA. Proteomic analysis of urine in medication-overuse headache patients: possible relation with renal damages. *The journal of headache and pain*. 2012;13:45-52.

2. Bellei E, Monari E, Cuoghi A, Bergamini S, Guerzoni S, Ciccarese M, Ozben T, Tomasi A and Pini LA. Discovery by a proteomic approach of possible early biomarkers of drug-induced nephrotoxicity in medication-overuse headache. *The journal of headache and pain*. 2013;14:6.

3. Biagianti B, Grazzi L, Gambini O, Usai S, Muffatti R, Scarone S and Bussone G. Orbitofrontal dysfunction and medication overuse in patients with migraine. *Headache*. 2012;52:1511-9.

4. Cevoli S, Marzocchi N, Capellari S, Scapoli C, Pierangeli G, Grimaldi D, Naldi F, Pini LA, Montagna P and Cortelli P. Lack of association between five serotonin metabolism-related genes and medication overuse headache. *The journal of headache and pain*. 2010;11:53-8.

5. Diener HC, Bussone G, Van Oene JC, Lahaye M, Schwalen S and Goadsby PJ. Topiramate reduces headache days in chronic migraine: a randomized, double-blind, placebo-controlled study. *Cephalalgia : an international journal of headache*. 2007;27:814-23.

6. Ferraro S, Grazzi L, Mandelli ML, Aquino D, Di Fiore D, Usai S, Bruzzone MG, Di Salle F, Bussone G and Chiapparini L. Pain processing in medication overuse headache: a functional magnetic resonance imaging (fMRI) study. *Pain medicine (Malden, Mass)*. 2012;13:255-62.

7. Ghiotto N, Sances G, Galli F, Tassorelli C, Guaschino E, Sandrini G and Nappi G. Medication overuse headache and applicability of the ICHD-II diagnostic criteria: 1-year follow-up study (CARE I protocol). *Cephalalgia : an international journal of headache*. 2009;29:233-43.

8. Grande RB, Aaseth K, Saltyte Benth J, Gulbrandsen P, Russell MB and Lundqvist C. The Severity of Dependence Scale detects people with medication overuse: the Akershus study of chronic headache. *Journal of neurology, neurosurgery, and psychiatry*. 2009;80:784-9.

9. Grande RB, Aaseth K, Benth JS, Lundqvist C and Russell MB. Reduction in medication-overuse headache after short information. The Akershus study of chronic headache. *European journal of neurology : the official journal of the European Federation of Neurological Societies*. 2011;18:129-37.

10. Imai N, Kitamura E, Konishi T, Suzuki Y, Serizawa M and Okabe T. Clinical features of probable medication-overuse headache: a retrospective study in Japan. *Cephalalgia : an international journal of headache*. 2007;27:1020-3.

11. Lundqvist C, Benth JS, Grande RB, Aaseth K and Russell MB. An adapted Severity of Dependence Scale is valid for the detection of medication overuse: the Akershus study of chronic headache. *European journal of neurology : the official journal of the European Federation of Neurological Societies*. 2011;18:512-8.

12. Lundqvist C, Grande RB, Aaseth K and Russell MB. Dependence scores predict prognosis of medication overuse headache: a prospective cohort from the Akershus study of chronic headache. *Pain*. 2012;153:682-6.

13. Munksgaard SB, Bendtsen L and Jensen RH. Detoxification of medication-overuse headache by a multidisciplinary treatment programme is highly effective: a comparison of two consecutive treatment methods in an open-label design. *Cephalalgia : an international journal of headache*. 2012;32:834-44.

14. Paemeleire K, Bahra A, Evers S, Matharu MS and Goadsby PJ. Medication-overuse headache in patients with cluster headache. *Neurology*. 2006;67:109-13.

15. Peres MF, Mercante JP, Guendler VZ, Corchs F, Bernik MA, Zukerman E and Silberstein SD. Cephalalgiaphobia: a possible specific phobia of illness. *The journal of headache and pain*. 2007;8:56-9.

16. Riederer F, Marti M, Luechinger R, Lanzenberger R, von Meyenburg J, Gantenbein AR, Pirrotta R, Gaul C, Kollias S and Sandor PS. Grey matter changes associated with medication-overuse headache: correlations with disease related disability and anxiety. *The world journal of biological psychiatry : the official journal of the World Federation of Societies of Biological Psychiatry*. 2012;13:517-25.

17. Rossi P, Faroni JV and Nappi G. Medication overuse headache: predictors and rates of relapse in migraine patients with low medical needs. A 1-year prospective study. *Cephalalgia : an international journal of headache*. 2008;28:1196-200.

18. Sances G, Galli F, Anastasi S, Ghiotto N, De Giorgio G, Guidetti V, Firenze C, Pazzi S, Quartesan R, Gallucci M and Nappi G. Medication-overuse headache and personality: a controlled study by means of the MMPI-2. *Headache*. 2010;50:198-209.

19. Zappaterra M, Guerzoni S, Cainazzo MM, Ferrari A and Pini LA. Basal cutaneous pain threshold in headache patients. *The journal of headache and pain*. 2011;12:303-10.

20. Zidverc-Trajkovic J, Pekmezovic T, Jovanovic Z, Pavlovic A, Mijajlovic M, Radojicic A and Sternic N. Medication overuse headache: clinical features predicting treatment outcome at 1-year follow-up. *Cephalalgia : an international journal of headache*. 2007;27:1219-25.
